# CT Diagnosis of an Abortion-Related Retroperitoneal Space Abscess

**DOI:** 10.4021/jocmr509w

**Published:** 2011-09-26

**Authors:** Spyridopoulos N Themistoklis, Vergadis Chrysovalantis, Argentos Stylianos, Kelekis L Nikolaos, Alexopoulou Efthymia

**Affiliations:** a 2nd Department of Radiology, Attikon Hospital, National and Kapodistrian University of Athens, Greece

## Abstract

A female patient with a history of recent abortion was transferred in critically ill condition in our hospital. CT imaging on emergency basis depicted an abscess in the right retroperitoneal space that included the head of fetus, along with rupture of the right lateral wall of uterus. Spiral CT enabled prompt diagnosis and therapy of this rare abortion-related complication.

**Keywords:**

CT-Spiral; Uterus; Complication; Rupture; Obstetrics

## Introduction

Surgical abortions are relatively safe procedures; although rare, severe, life-threatening complications may occur. Clinical symptoms and signs should be estimated by clinicians and computed tomography (CT) imaging should be requested. The implementation of optimized CT study protocols along with images’ reconstruction allows a detailed imaging of pelvic and abdomen structures. CT findings are usually diagnostic for uterine rupture in an emergency setting.

## Case Report

A 31-year-old white woman was transferred from an outside hospital and referred to the Emergency Unit of our hospital with fever, abdominal pain and metrorrhagia. Her otherwise unremarkable medical history revealed a recent pregnancy termination (10 days earlier) at 17 gestational weeks, due to high risk of a chromosome abnormality of the fetus. According to her medical record, labour induction was firstly attempted with vaginally applied prostaglandin agents; however, the treating physicians proceeded to surgical abortion and laparotomy.

**Figure 1:**
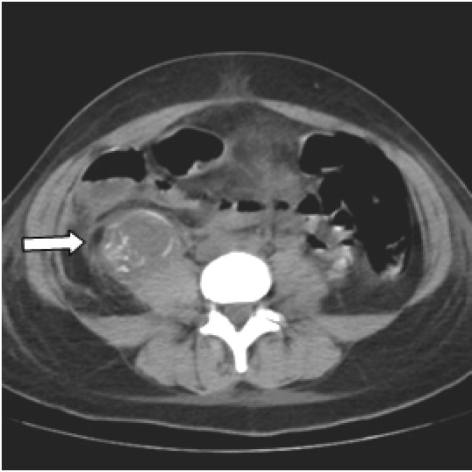
Unenhanced lower abdomen CT scan shows a retroperitoneal hypodense lesion with internal hyperdense bony elements, in contact with the right psoas muscle (arrow).

**Figure 2:**
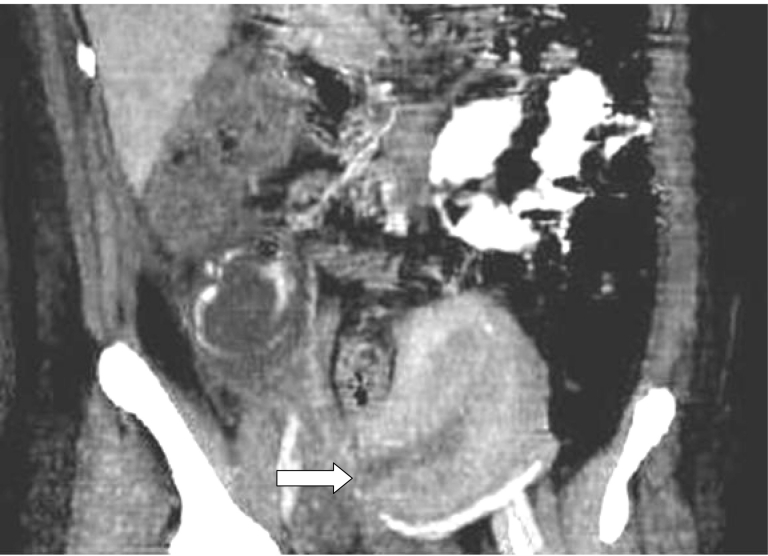
Enhanced reconstructed CT image (oblique coronal) shows the inconsistency of the right lateral wall of the myometrium (arrow), a finding indicative of perforation or rupture. The retroperitoneal lesion, with air bubbles and peripheral enhancement is also visible.

Clinical and laboratory tests revealed tachypnea, tachycardia, leukocytocis and severe anaemia. An urgent abdomen CT scan was performed. Unenhanced axial CT scans of the abdomen revealed a circumscribed fluid collection with internal hyperdense (calcified or bony) elements in the right retroperitoneal region. Viewing images with different window settings and based on the patient’s history we were highly suspicious that the bony formation in the right retroperitoneal space represented the skull of the fetus’ head (skull, orbital and nasal bones, right zygoma, as presented in (Fig. 1). Peripheral enhancement after iv contrast administration and the presence of air bubbles, were suggestive of an abscess. Enhanced scan reconstructions - thick oblique coronal multiplanar reformations (thick MPR) - showed additionally inconsistency of the right lateral wall of the uterus, indicative of perforation or rupture (Fig. 2).

The patient underwent an explorative laparotomy that revealed perforation of the right lateral wall of the uterus and the formation of an abscess in the right retroperitoneal space around the fetus’ head. A total hysterectomy was performed and the patient was discharged after 10 days.

## Discussion

Abortion is considered to be safe procedure; older age, multiparity, and advancing gestational age increase the risk of abortion-related complications [1]. Complications in failed surgical abortions are uncommon; however, if large amounts of retained tissue are left, the patient is at risk of bleeding, infection and occasionally sepsis [2]. Uterine rupture is unreported in first-trimester abortion; however, it occurs on occasion in both instrumental and labor induction abortions in the second trimester [3]. Uterine perforation may be unrecognized [4]. The most frequent site of myometrial perforation with all types of intrauterine surgery is the relatively avascular anterior or posterior midline surface of the uterus; most of these injuries are treated conservatively. Perforations of the lateral walls of the uterus are more problematic, occur beyond the first trimester and result in acute abdominal pain or metrorrhagia.

CT is an ideal modality for urgent imaging in suspicion of uterine rupture. Disruption of regular continuity of uterine walls may be the first pathologic CT finding. Abrupt breaking of homogeneously attenuated uterine walls and extrusion of placental contents are diagnostic for the pathology [5]. Low density or heterogeneous fluid with or without gas bubbles may be identified in the endometrial cavity or into the peritoneal cavity in case of uterine rupture. Ultrasonography may reveal a heterogeneous, irregular echostructure that involves the entire thickness of uterine myometrium, extrusion of hematoma, or a heterogeneous mass between uterus and urinary bladder [6]. MRI may be a better modality in discriminating soft tissues, but it is less available in an emerging setting [7].
